# Assessing current clinical eruption stage of mandibular third molars by dental panoramic radiography

**DOI:** 10.2340/aos.v83.40477

**Published:** 2024-04-23

**Authors:** Tommi Vesala, Irja Ventä, Johanna Snäll, Magdalena Marinescu Gava, Marja Ekholm

**Affiliations:** a Department of Oral and Maxillofacial Diseases, Faculty of Medicine, University of Helsinki, Helsinki, Finland; b Department of Oral and Maxillofacial Diseases, Helsinki University Hospital, Helsinki, Finland; c Finnish Student Health Service, Helsinki, Finland; d Turku University Hospital and University of Turku, Turku, Finland

**Keywords:** Molar, Third, Radiography, Panoramic, Tooth Eruption, Tooth, Unerupted, Diagnosis, Oral

## Abstract

**Objective:**

We examined whether dental panoramic radiography (PAN) can be used to identify the clinical stage of eruption of mandibular third molars at the time of radiological examination.

**Materials and methods:**

Cross-sectional data included records from clinical oral examination and PANs of university students. In the retrospective analysis of 345 mandibular third molars in 189 participants (20% men, 80% women; mean age 20.7 years; standard deviation [SD] ± 0.6), clinical stages of eruption were compared with their radiographic depth in bone, inclination, and root development. Statistics included χ^2^, Mann-Whitney U tests, and logistic regression.

**Results:**

Significant (*p* < 0.001) predictor variables for assessing the clinical stage of eruption were radiographic depth in bone and inclination. All teeth radiologically at a depth of the cementoenamel (CE) junction of the neighbouring second molar or deeper were clinically unerupted. Above the CE junction, 80% of vertical and 97% of distoangular teeth were connected to the oral cavity, and 82% of mesioangular and 69% of horizontal teeth were clinically unerupted.

**Conclusion:**

All teeth below or at the CE junction are clinically unerupted. Above the CE junction, stage of eruption should be assessed together with the inclination, but horizontally inclined teeth are recommended to be verified clinically.

## Introduction

Need for extraction of a third molar is based on clinical oral examination and radiological imaging, mainly dental panoramic radiography (PAN) [1–3]. The main finding leading to extraction may appear in either clinical or radiological examination, but both are needed to support the treatment decision. The assessment considers whether the removal is appropriate for the patient, weighing possible advantages and disadvantages as well as risk of complications. However, in consultation situations, e.g., from other clinicians, the clinical examination of the oral cavity may be partial or missing. For example, a medical physician may refer a patient to PAN imaging due to symptoms or findings in the head and neck area or for infection foci evaluation. Thus, a radiologist is obliged to interpret the third molars on the PAN without knowing the exact clinical status. The research question of the present work arose from this practical need of a radiologist.

Unerupted third molars embedded entirely in the bone structure are not interrelated with the bacterial flora of the oral cavity, and therefore, infrequently cause marked pathologies [4, 5]. However, when perforation of marginal cortex and gingiva is established, the third molar is exposed to the oral bacterial flora. Partially erupted third molars are risk factors for several pathologies such as pericoronitis, periodontal disease, caries, bone loss, and resorption of the adjacent second molar [6–9]. Thus, in situations where patient is referred to PAN imaging, e.g., due to infection foci evaluation, it would be beneficial to radiologically recognise teeth connected to the oral cavity and subsequently susceptible to oral microbiological flora and infection.

Partially erupted third molars can be identified easily in clinical oral examination, either visually or with a probe from the distal pocket of the second molar. However, based only on a PAN the assessment of clinical stage is challenging, especially for third molars that have perforated just the marginal cortex. Generally accepted radiological criteria for estimating current clinical stage of eruption do not exist. Based on radiographic measurements, the literature search yields studies predicting future eruption of third molars but none assessing clinical stage of eruption at the time of radiological examination [10]. Thus, it is appropriate to clarify the clinical relevance of radiographic findings of mandibular third molars.

The aim of this study was to examine whether the clinical stage of eruption of mandibular third molars can be assessed from PAN at the time of radiological examination. The hypothesis was that the PAN can be used to identify if a connection exists clinically between the mandibular third molar and the oral cavity.

## Materials and methods

### Study design

A retrospective study was designed to compare radiographic characteristics with clinical eruption stages of mandibular third molars in a 21-year-old student cohort. An existing cross-sectional material was utilised from a previous study gathered at the Finnish Student Health Service (FSHS), Helsinki, Finland in 2002 [11, 12]. At that time at the FSHS, it was routine to invite all first-year students to a free dental examination. The 21-year-old cohort was recruited to a more detailed oral health study and after the clinical oral examination they were invited to participate also in the radiography. The study cohort comprised all students born in Helsinki in 1981 or 1982 who had started their first-year studies at any faculty at the University of Helsinki in 2001 and were living in Helsinki at that time. The narrow age range and similar background (place of birth and present residential area) were chosen to avoid possible bias of the material. The present study included students who had participated in 2002, whose records from clinical oral examination and PAN were available for the present analysis, and who had at least one mandibular third molar at the time the PAN was taken.

### Study variables

The data for the present study included records from clinical oral examinations and PANs. The following variables were recorded from the data: age and sex as explanatory variables, identification of each mandibular third molar, its clinical stage of eruption, radiographic depth in the bone, inclination, and stage of root development (Table 1).

**Table 1:** Definitions of study variables of mandibular third molars, including clinical stage of eruption and radiographic characteristics on panoramic radiograph.

Characteristic	Category	Definition
Clinical stage of eruption		
	Unerupted	Clinically invisible and cannot be probed
	Connected to oral cavity	Can be felt with a probe in the distal pocket of a second molar Part of the occlusal surface visible Occlusal surface completely visible Crown partially visible Crown completely visible
Radiographic characteristics		
Depth of the most cranial point of the crown in alveolar bone (Figure 1)	A	Below the marginal cortex
	B	In the marginal cortex but not perforating it
	C	Has perforated the marginal cortex: location below or at cementoenamel junction of the second molar
	D	Has perforated the marginal cortex: location between cementoenamel junction of the second molar and occlusal surface
	E	Located at the occlusal surface
Inclination	Vertical	0° – 10°
	Distoangular	-1° – -70°
	Mesioangular	(+11° – +70°)
	Horizontal	Mesiohorizontal (> +70°) and transversal
Stage of root development [13]	Incomplete	Apices of roots not closed
	Finished	Apices closed

The primary outcome variable was the current stage of clinical eruption of the mandibular third molar. The clinical stage was recorded according to the extent of the crown clinically visible in the oral cavity. Presence of invisible third molars was ascertained with a probe from the distal pocket of the second molars.

**Figure 1:**
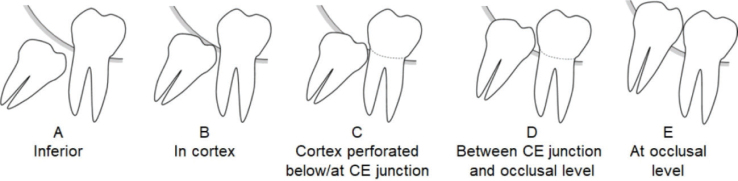
Radiographic depth of mandibular third molar in alveolar bone as the location of the most cranial point of the crown of the tooth in relation to the marginal cortex and the neighbouring second molar. CE = cementoenamel.

Predictor variables were radiological findings on panoramic radiographs. The primary predictor variable was the depth of the tooth in the alveolar bone, and it was defined as the most cranial point of the mandibular third molar crown in relation to the marginal bone cortex and the neighbouring second molar (Figure 1). Secondary predictor variables were inclination of the tooth and stage of root development. Inclination of the tooth was measured and grouped as vertical, distoangular, mesioangular, mesiohorizontal, and transversal. The stage of root development was grouped as incomplete or finished.

Panoramic radiographs were obtained with Planmeca Promax 2D (Helsinki, Finland) with values of 64–68 kV and 6.3–10 mA depending on the participant’s size. Exposure time was 15.8 s. The PANs were examined by one of the authors at the facilities of the FSHS on a viewing screen and with an SDI X-ray reader (https://doi.org/10.1038/sj.bdj.4811380) in dimmed room lightning. After examination of all radiographs, 11% (*n* = 23) of randomly selected radiographs were examined a second time after 2 weeks to approximate intra-examiner reproducibility.

### Statistical analysis

The observational unit was a mandibular third molar. In the analysis, the clinical stages of eruption were dichotomised as unerupted teeth and all the rest were labelled as connected to the oral cavity, where the latter included all visible teeth and those that could be probed from the distal pocket of the second molar.

First, the radiographic depths of the tooth in the alveolar bone and the clinical stages of eruption were cross-tabulated. Second, binary logistic regression analysis (including sensitivity, specificity, and odds ratios [ORs] with their 95% confidence intervals [CIs]) was used to identify the independent variables that had a statistically significant effect on the clinical stage of eruption. In the regression analysis, best ORs and fit of model were sought by combination of categories. Finally, the impact of inclination on the clinical stage of eruption was assessed by cross-tabulation. Differences among various subgroups were evaluated using χ^2^ test for frequencies and Mann-Whitney U test for means of independent groups. Analyses were performed using Statistical Package for the Social Sciences (SPSS) Statistics, version 27 (IBM Corporation, Armonk, NY, USA).

### Ethical considerations

The original study protocol for clinical and radiographic examinations was approved by the FSHS institutional review board in 2002. The Helsinki Declaration guidelines were followed, and each subject participated voluntarily in the oral health examination. According to European Commission guidelines for radiation protection, routine radiography without patient’s history and clinical examination is unacceptable practice [1]. Thus, an existing radiographic material was reused for the present analysis. The Finnish Social and Health Data Permit Authority (Findata) granted permission for the secondary use of this health care data (THL/4680/14.02.00/2020). Permission to reuse the existing material was also obtained from the FSHS. Based on the General Data Protection Regulation (GDPR) of the European Parliament concerning personal data, only results of frequencies greater than 5 are presented, and therefore, some combinations of categories in the analyses were made.

## Results

**Figure 2:**
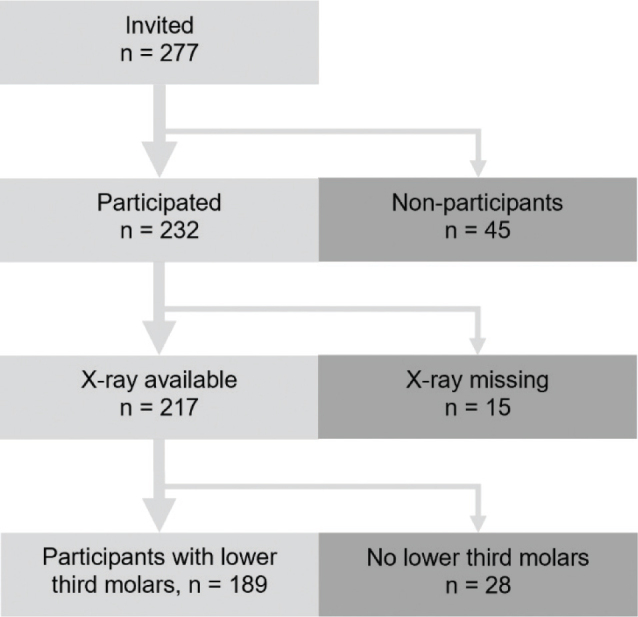
Flow diagram of included and excluded participants.

Of the 277 invited students, 232 (84%) participated in the oral health examination. The most common reasons for not participating were no-show (35%), living abroad or studying elsewhere (19%), and military service (16%). Of the participants, 15 persons were excluded for missing PAN images in the present examination (Figure 2).

Of the 217 persons with panoramic radiographs, at least one mandibular third molar was observed in 189 persons, of whom 20% were men and 80% women. The mean age of these persons was 20.7 years (standard deviation (SD) ±0.6 years, range 19.7–21.7 years). The number of mandibular third molars in these 189 persons was 345 (174 on the left side, 171 on the right side) (Table 2). The included participants (*n* = 189) and the excluded participants (*n* = 43) with missing radiographs or missing mandibular third molars did not differ significantly by sex (χ^2^ = 1.26; df = 1; *p* = 0.261) or age (Mann-Whitney U = 4,160; *p* = 0.808). Regarding intra-examiner reliability of the observations about the PANs, the kappa values were 0.95 for depth of the tooth in the bone, 0.94 for stage of root development, and 1.00 for other measurements. Kappa values between 0.81 and 1.00 were considered very good.

**Table 2:** Distribution of mandibular third molars in 189 persons according to clinical stages of eruption, by sex.

Sex	Unerupted		Felt with probe or partially visible		Crown completely visible		Total
	*n*	%	*n*	%	*n*	%	*n*
Male	35	50	27	39	8	11	70
Female	150	55	106	38	19	7	275
Total	185	54	133	38	27	8	345

**Figure 3:**
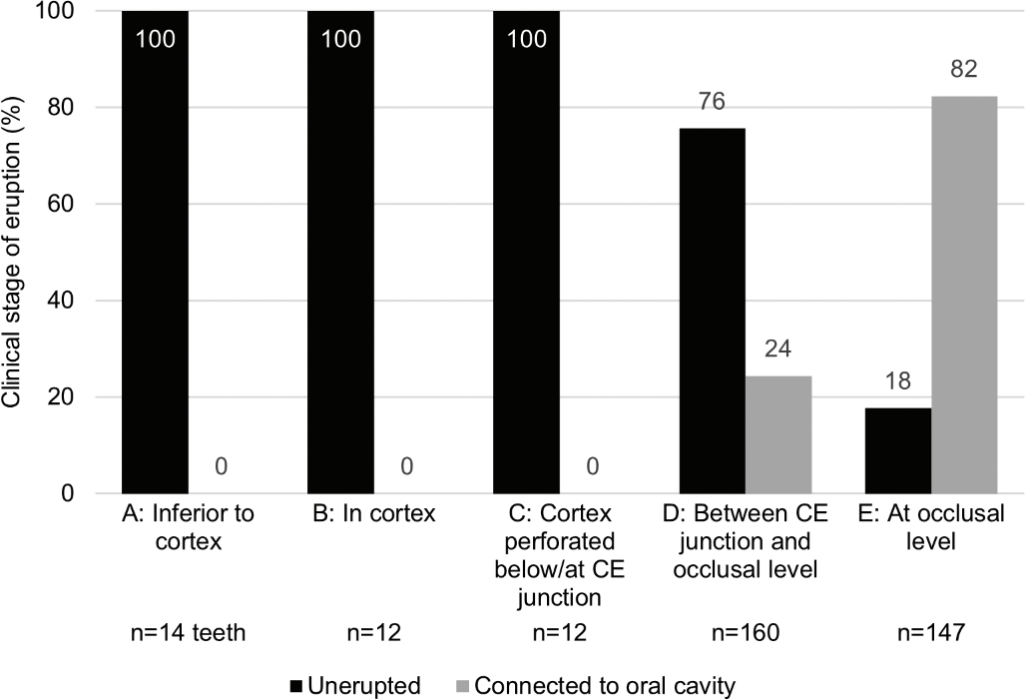
Relationship of radiographic depth in alveolar bone (horizontal axis) to clinical stage of eruption (vertical axis) of mandibular third molars. Clinical stage of eruption dichotomised as unerupted or connected to oral cavity. Number of third molars (total *n* = 345) in each radiographic category is revealed at the bottom of the figure. CE = cementoenamel.

The relationship of the radiographic depth in alveolar bone to the clinical stage of eruption of mandibular third molars is presented in Figure 3. All teeth radiologically at a depth of cementoenamel (CE) junction of the neighbouring second molar or deeper were clinically unerupted. Above the CE junction, the distributions were split.

As explained by logistic regression analysis of the teeth above the CE junction (*n* = 307 teeth), significant (*p* < 0.001) predictors for clinical eruption were the radiographic depth of the tooth in alveolar bone and inclination of the tooth (Table 3). A third molar was significantly more likely to have a connection to the oral cavity if it was located at the occlusal level compared with a location below occlusal level (OR 10.9; 95% CI 5.28–22.54). Vertical and distoangular inclinations also significantly more likely had a connection to the oral cavity than mesioangular, mesiohorizontal, and transversal positions (OR 21.81; 95% CI 10.49–45.34). Age, sex, and stage of root development were not significant predictors in the model. The logistic regression model was statistically significant (χ^2^ = 212.43; df = 5; *p* < 0.001), explained 67% (Nagelkerke R^2^) of the variance in the clinical state of eruption, and correctly classified 83% of cases. Sensitivity (true positives) of the model was 87% and specificity (true negatives) 79%.

**Table 3:** Logistic regression analysis of mandibular third molars above the CE junction (classes D and E in Figure 1, *n* = 307 teeth) predicting likelihood of a third molar having clinically a connection to the oral cavity.

Characteristic	*P*	OR	95% CI
Age	0.509	1.24	0.66; 2.32
Sex			
[Female]			
Male	0.798	1.12	0.47; 2.71
Depth in alveolar bone			
[Between CE junction and occlusal level, Group D]			
At occlusal level, Group E	< 0.001	10.91	5.28; 22.54
Inclination			
[Inclined]^a^			
Vertical and distoangular	< 0.001	21.81	10.49; 45.34
Stage of root development			
[Incomplete]			
Finished	0.115	1.82	0.86; 3.85

OR odds ratio, CI confidence interval.

Hosmer and Lemeshow’s test for the model indicated good fit (χ^2^ = 4.45; df = 8; *p* = 0.815).

a Mesioangular, mesiohorizontal, and transversal teeth.

Reference group is in square brackets.

In the cross-tabulation between inclinations and the clinical eruption stages of teeth above the CE junction (*n* = 307 teeth), the difference of frequencies was statistically significant (χ^2^ = 148.27; df = 3; *p* < 0.001). Vertical teeth above the CE junction were connected to the oral cavity in 80% (95% CI 71.64–88.82) of cases and distoangular teeth in 97% (95% CI 92.37–100) of cases. Mesioangular teeth were clinically unerupted in 82% (95% CI 76.05–88.74) and horizontal teeth in 69% (95% CI 43.24–94.26) of cases.

## Discussion

The purpose of the study was to identify radiographic characteristics which could be used to assess the current clinical stage of eruption of mandibular third molars. Supporting the hypothesis, radiographic depth in the bone and inclination of the tooth proved to be useful.

Our results showed that among the 21-year-old adults all mandibular third molars at a depth of the CE junction or deeper (locations A, B, and C in Figure 1) were clinically unerupted. Distribution of teeth above the CE junction was split, and therefore, additional radiographic variables were needed to assess the clinical stage. Logistic regression analysis indicated that a practical variable for this purpose was inclination. Of all mesioangular, horizontal, vertical, and distoangular third molars above the CE junction, the proportion of teeth connected to the oral cavity was 18, 31, 80, and 97%, respectively. These findings suggest a useful means to assess clinical characteristics of third molars on a PAN at the time of radiological examination.

Due to the scarcity of similar articles, it is difficult to compare our findings with published studies. In the literature, a common topic is to identify radiographic characteristics of third molars predisposing to pericoronitis. For example, in a Swedish study on 666 patients at oral and maxillofacial surgery clinics, mandibular third molars in vertical or distoangular positions infrequently had full tissue coverage compared with other angulations, and thus, were prone to pericoronitis [4]. In a Spanish study of 165 patients undergoing extraction of mandibular third molars, a high correlation emerged between mandibular third molars located at the occlusal level and partial or no mucosal coverage [5]. These results are in line with our findings that vertical and distoangular third molars above the CE junction were mostly connected to the oral cavity. However, other parameters were not comparable in these studies. Another common topic in the literature is to use the same radiographic characteristics of third molars as in our study to assess future eruption (some years later), not the current stage of eruption at the time that the PAN was taken [10]. While findings of such studies are not directly comparable with ours, they do suggest that third molars in a certain inclination and location are prone to erupt.

The narrow age distribution in our study (range 19.7–21.7 years) corresponds to the typical age of third molar eruption [14]. Therefore, it is understandable that the proportion of deep locations of third molars (A, B, and C in Figure 1) was small relative to superficial locations (D and E). The number of third molars in deep locations would probably have decreased in the following years, and an analysis such as ours would not have been possible in older age groups. Furthermore, the sex distribution in our study was female-oriented. This is explained by the fact that around 64% of bachelor’s and 68% of master’s degree students at the University of Helsinki are women. Also, women participated in the original examination more actively than men (88% vs. 74%) [11]. However, logistic regression analysis showed that sex was not an important predictor.

The importance of our findings is emphasised in situations where a statement about mandibular third molars is provided based on a PAN alone, without precise clinical information. This problem may be faced by a radiologist writing a statement, an expert body making an insurance judgement, or in consultation situations at the request of other clinicians. For example, in patients with forthcoming antiresorptive or immunosuppressive medication or radiation treatment of the jaws, partially erupted third molars among other things are considered infection foci [15, 16], which should be identified from the PAN. In our study, all third molars in deep locations (A, B, or C) had no connection with the oral cavity, and thus, a low probability of upcoming pathological conditions. On the other hand, among locations above the CE junction, the accuracy of assessment of the clinical stage of eruption was high but not complete. Therefore, a recommendation for further clinical examination of such patients would be appropriate, especially for those with horizontal mandibular third molars.

Our findings on the method to assess clinical characteristics of third molars based on a PAN are likely to be applicable to all countries. According to the logistic regression analysis, age, sex, and development of the root were not significant predictors in the assessment, suggesting that this method could be suitable for all ages and sexes. However, the method is only partially applicable to patients with missing second molars since they lack a reference point. In elderly patients, it should also be considered whether thinning of the oral mucosa can affect the evaluation of clinical status [17], and therefore, third molars at the occlusal level may be clinically connected to the oral cavity more frequently than in our 21-year-old cohort.

A limitation of the study was the small number of mandibular third molars located deep in the bone. This limitation could have been tackled had the study included more participants younger than the mean age of 20.7 years. A strength of the study was the detailed examination of the clinical stage of eruption at baseline. Another strength was that the students participated voluntarily in a routine oral health examination and were not patients referred to surgical extraction of third molars, where such diagnoses as pericoronitis and cysts with bone resorption might have blurred the integrity of the marginal bone cortex.

In summary, it is possible to assess the current stage of clinical eruption of mandibular third molar from a PAN with a reasonable degree of certainty. Based on our findings, the following conclusions and recommendations for clinical practice can be drawn regarding mandibular third molars in young adults:

1. All teeth in deep bone locations (below or at the CE junction) are clinically unerupted.
2. More superficial locations (above the CE junction) should be assessed together with the inclination of the tooth.
3. Vertical and distoangular teeth above the CE junction are most likely connected to the oral cavity (80–97% of cases here).
4. Mesioangular teeth above the CE junction are often clinically unerupted (82% of cases here).
5. Horizontal teeth above the CE junction are recommended to be verified clinically.
